# Bumblebees can detect floral humidity

**DOI:** 10.1242/jeb.240861

**Published:** 2021-06-24

**Authors:** Michael J. M. Harrap, Natalie Hempel de Ibarra, Henry D. Knowles, Heather M. Whitney, Sean A. Rands

**Affiliations:** 1 School of Biological Sciences, University of Bristol, Bristol, BS8 1TQ, UK; 2 Centre for Research in Animal Behaviour, School of Psychology, University of Exeter, Exeter, EX4 4QG, UK; 3 Natural Resources Wales, Maes Newydd, Llandarcy, Neath Port Talbot, SA10 6JQ, UK

**Keywords:** Behaviour, Floral Display, Multimodal cues, Learning, Bumblebees, Angiosperms, Flower, Pollination

## Abstract

Floral humidity, a region of elevated humidity in the headspace of the flower, occurs in many plant species and may add to their multimodal floral displays. So far, the ability to detect and respond to floral humidity cues has been only established for hawkmoths when they locate and extract nectar while hovering in front of some moth-pollinated flowers. To test whether floral humidity can be used by other more widespread generalist pollinators, we designed artificial flowers that presented biologically relevant levels of humidity similar to those shown by flowering plants. Bumblebees showed a spontaneous preference for flowers that produced higher floral humidity. Furthermore, learning experiments showed that bumblebees are able to use differences in floral humidity to distinguish between rewarding and non-rewarding flowers. Our results indicate that bumblebees are sensitive to different levels of floral humidity. In this way floral humidity can add to the information provided by flowers and could impact pollinator behaviour more significantly than previously thought.

## Abstract (precis)

**Highlighted Article:** Bumblebees show a preference for elevated floral humidity and can learn to distinguish flowers that differ in floral humidity levels.

## INTRODUCTION

Floral humidity, an area of elevated humidity within the headspace of the flower, has been demonstrated to occur in a number of flower species (Corbet et al., 1979; Nordström et al., 2017; von Arx et al., 2012). Floral humidity is created by a combination of nectar evaporation and floral transpiration (Azad et al., 2007; Corbet et al., 1979; Harrap et al., 2020a; von Arx et al., 2012), although the contribution of these two influences may vary between species. Transects of the flower headspace of 42 species found 30 (71%) produce floral humidity of an intensity greater than would be expected from any conflating environmental humidity sources (Harrap et al., 2020a) (such as the minimal humidity differences due to uneven air mixing in the sampling room, or humidity produced by water within the capped horticultural tubes that flowers were mounted in during sampling). The intensity of floral humidity produced by flowers, represented by 
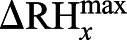
 (the average peak difference in relative humidity in the flower species' headspace compared with the background), reached up to 3.71% (in *Calystegia sylvatica*). Floral humidity occurs widely and varies between species (Harrap et al., 2020a) and does not appear to be limited to species visited by a particular group of pollinators (Harrap et al., 2020a): elevated floral humidity intensity has been observed in flowers pollinated primarily by moths (von Arx et al., 2012), flies (Nordström et al., 2017) and bees (Corbet et al., 1979).

Whether such variations in floral humidity can be used as a foraging cue is poorly understood, and has only been demonstrated in a single pollinator species, *Hyles lineata*, a hawkmoth frequently pollinating *Oenothera caespitosa* (von Arx, 2013; von Arx et al., 2012). It was demonstrated that *H. lineata* shows a preference for artificial flowers producing floral humidity comparable to that produced by *O. caespitosa*, over those at ambient humidity. Investigation of the capacity of pollinators other than *H. lineata* to respond to floral humidity is limited (von Arx, 2013), with non-experimental observations that flies may use floral humidity in addition to other floral display traits produced within Indian alpine environments (Nordström et al., 2017). Given that floral humidity is present in many flower species, as recently measured by Harrap et al. (2020a), it is most likely that floral humidity is regularly encountered as part of flowers' multimodal displays by a wide range of generalist pollinators and influences their foraging behaviours.

Sensitivity to environmental (non-floral) humidity is well reported in insects (Enjin, 2017; Havukkala and Kennedy, 1984; Kwon and Saeed, 2003; Lin et al., 2014; Liu et al., 2007; McCall and Primack, 1992; Peat and Goulson, 2005). Honeybees *Apis mellifera* respond to humidity levels within the nest, regulating humidity to different levels in different parts of the nest (Human et al., 2006; Nicolson, 2009). Elevated humidity triggers nest ventilation behaviours in bees such as fanning nest structures, and low humidity encourages behaviours that increase nest humidity by the evaporation of nectar water or water collection (Abou-Shaara et al., 2017; Ellis et al., 2008; Human et al., 2006). Biting flies and mosquitoes are thought to respond to humidity given off by their host organisms, among other cues (Chappuis et al., 2013; Olanga et al., 2010; Smart and Brown, 1956). Mosquitoes also make use of humidity to locate still-water oviposition sites (Okal et al., 2013). Furthermore, following the presentation of sugar water droplets that touch their antenna, restrained honeybees have been seen to show a proboscis extension response to droplets of water placed near (but not touching) the antenna (Blenau and Erber, 1998; Kuwabara, 1957; Mercer and Menzel, 1982). This is probably a response to the water vapour (i.e. humidity) given off by the droplet, suggesting that bees can be conditioned based on humidity to some degree (Blenau and Erber, 1998; Kuwabara, 1957; Mercer and Menzel, 1982). Taken together with the presence of hygrosensitive (humidity-detecting) sensilla in many pollinating insects, this suggests that pollinator groups other than hawkmoths possess the necessary sensory mechanisms to detect and respond to humidity cues and signals in the context of foraging on flowers.

The presence of a hygrosensitive antennal sensillum, the ceolocapitular sensillum (Yokohari, 1983; Yokohari et al., 1982), has been reported for bees (Ahmed et al., 2015; Tichy and Kallina, 2014; Yokohari, 1983; Yokohari et al., 1982); these sensilla are common and show a wide distribution across the antenna in *Bombus* bumblebees (Fialho et al., 2014). This may allow bumblebees to show higher humidity sensitivity (Fialho et al., 2014), although the exact mechanism by which these ceolocapitular sensilla detect humidity is uncertain (Enjin, 2017; Tichy and Kallina, 2010). Insects always possess two types of humidity-sensitive cells within ceolocapitular sensilla: dry cells, which respond to a lack of humidity; and moist cells, that respond to its presence (Yokohari, 1983; Yokohari et al., 1982). In addition to signalling based on the humidity at a given instant, moist and dry cells signal with a greater frequency in response to the rate of humidity changes (Tichy, 2003; Tichy and Kallina, 2010, 2014). Insects can therefore detect both the humidity at a given time, and also the rate and direction of humidity changes, getting drier or moister. The levels of humidity produced by flowers are similar to the sensitivity range reported in *Apis mellifera* (Harrap et al., 2020a; Tichy and Kallina, 2014), suggesting that floral humidity differences could feasibly be detected and used by pollinators while foraging.

We investigated the capacity of bumblebees *Bombus terrestris* to detect and respond to artificial flowers producing floral humidity at levels comparable to the floral humidity detected in real flowers. Furthermore, in these experiments we explored how floral humidity may affect bumblebee foraging behaviours. We ask whether floral humidity cues influence bumblebee spontaneous flower choices. Additionally, as pollinators can learn to associate differences between flowers in various floral traits to distinguish more rewarding flowers from less-rewarding flowers, such as colour (Gumbert and Kunze, 2001; Streinzer et al., 2009), scent (Daly et al., 2001; Galen and Newport, 1988), floral temperature (Dyer et al., 2006; Whitney et al., 2008), floral texture (Kevan and Lane, 1985), electrostatic properties (Clarke et al., 2013) and patterning of these signals (Hempel de Ibarra et al., 2015; Harrap et al., 2017, 2020b; Lawson et al., 2018; Whitney et al., 2009a), we also conducted differential conditioning experiments to investigate the capacity of bumblebees to learn to distinguish rewarding and non-rewarding flowers based on differences in floral humidity.

## MATERIALS AND METHODS

Responses to floral humidity were tested in lab conditions using captive (female worker) bumblebees, *Bombus terrestris audax* (Harris 1776) obtained from Biobest (Westerlo, Belgium via Agralan, Swindon, UK). Bumblebees are an appropriate choice of forager to test responses to floral humidity as they visit a wide range of species, including many of those found to produce different levels of floral humidity by Harrap et al. (2020a). For example, bee pollinators are known to forage on species throughout the range of floral humidity observed (Harrap et al., 2020a), such as: *Calystegia sylvatica* (
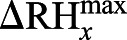
=3.71%); *Eschscholzia californica* (
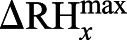
=3.24%); *Scabiosa* (
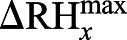
=1.36%); *Osteospermum* (
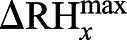
=1.20%); *Papaver cambricum* (
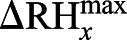
=0.29%); *Papaver rhoeas* (
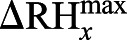
=0.29%); and *Fuchsia* (
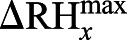
=0.05%). Bumblebee colonies were kept within a humidity- and temperature-controlled lab, maintained at 30% and 21°C, respectively. Colonies were each attached to a flight arena (dimensions 72×104×30 cm, width×length×height) via a clear access tube that could be closed off to control bee access. This flight arena had a clear Perspex lid and six doors allowing experimenter access. Arenas were illuminated by multiple daylight bulbs (Sylvania Activa 172 Professional 36 W fluorescent tubes, Havells-Sylvania Germany GmbH, Erlangen, Germany). Outside of experimental trials, bees were fed pollen directly to the colony (provided 3 times a week) and 30% sucrose solution *ad libitum* within the flight arena, which they could access freely outside of trials. This sucrose solution was provided on artificial flowers, gravity feeders and PCR racks. These artificial flowers provided to bees outside of trials were different but similar in appearance to the artificial flowers used in experiments (detailed below), being made from the same specimen jars (Thermo Scientific Sterilin, PS 60 ml, with white plastic lids) with upturned Eppendorf tubes (Hamburg, Germany) stuck to the lid as feeding wells containing sucrose solution. These artificial flowers did not produce the test cues under investigation within our experiments (here floral humidity). Outside of using these flowers there was no further ‘pretraining’ step taken before bee trials. Foraging bees began the experimental trials naive to the artificial flowers used in experiments and floral humidity cues, but had experience foraging in the flight arenas on artificial flowers. Foraging bees were marked with non-toxic paint to allow identification of individual foraging bees for experiments. Further details of bee husbandry, marking for identification and the flight arena are described elsewhere (Harrap et al., 2017, 2019, 2020b; Pearce et al., 2017; Lawson et al., 2018). No ethical permissions were required for the experiments involving bumblebees, but the experiments were conducted according to ASAB/ABS guidelines.

We created two types of artificial flower that produced artificial floral humidity comparable in intensity and structure observed on natural flowers. In ‘active’ flowers, elevated humidity was created by pumping humid air into the flower, whereas in ‘passive’ flowers, a wettable sponge was placed within the flower to generate elevated floral humidity. The two types of artificial flowers allowed us to observe whether responses of bees were the same independently of how floral humidity was generated. Both types of flowers had two variants that varied in the level of humidity they produced. The ‘humid’ variant produced elevated humidity in the proximity of the flower's top, whilst in the ‘dry’ artificial flower variant, floral humidity was lower. To reward the bees, a well (created from the lid of an Eppendorf tube) in the centre of the artificial flowers provided a drop of sugar solution in rewarding flowers, and a drop of water in non-rewarding flowers. All artificial flowers were dry to the touch (to avoid conflating responses to wet flower surfaces) and were designed to not differ in temperature or other characteristics bees would show a foraging response to.

### Artificial flower design: active flowers

**Fig. 1.**
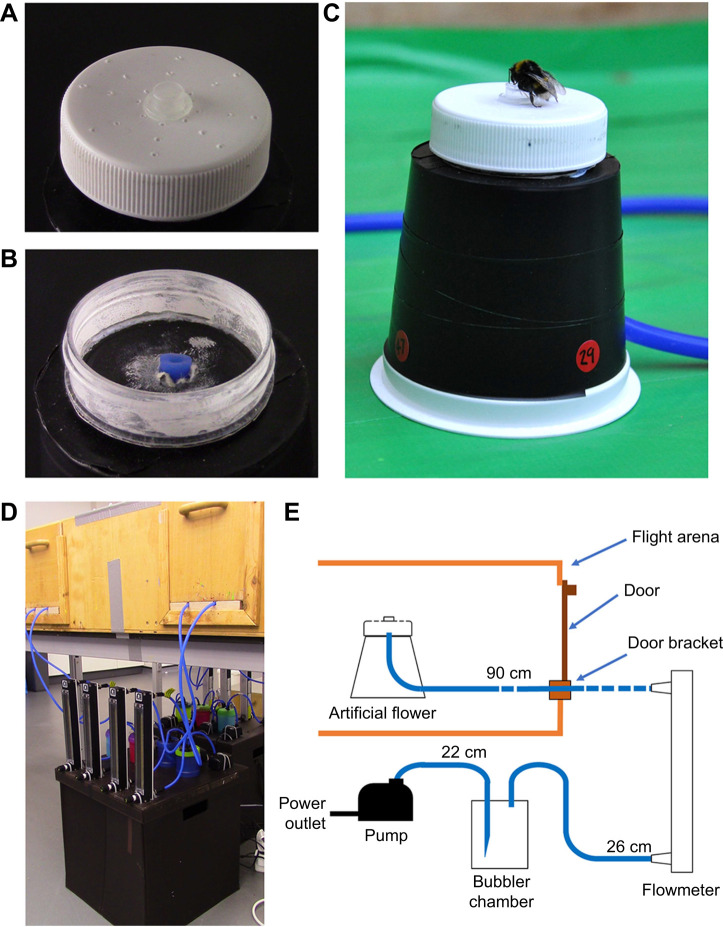
**The active artificial flowers used in bumblebee experiments.** (A) The artificial flower head. Note the holes on the flower head for air to escape. (B) The flower head with the head unscrewed, allowing the chamber under and pipe entry point to be seen. (C) A bumblebee feeding from active humidity flower as they appear in the flight arena. (D) The pump–bubbler–rotameter assembly installed below the flight arena. Note the rubber tubes entering the arena through brackets below the doors. (E) A diagrammatical representation of each artificial flower and its pump mechanism and how it is installed through the flight arena through a door bracket. Rubber tubes are represented by blue lines connecting components; the lengths of tubes are given above each tube.

The active flowers were similar to the hawkmoth-flower design used by von Arx et al. (2012), but were adapted to better suit bumblebee foraging behaviour. In this way, active flowers allowed us to test bee responses to a stimulus produced in a manner comparable to that study. These artificial flowers had a flower top with small holes (Fig. 1A) to a chamber below the flower head (Fig. 1B). This chamber was connected by 6 mm external, 4 mm internal diameter airline tubing (MARINA blue airline, Hagen, Mansfield, MA, USA) to a pump assembly outside the foraging arena (Fig. 1D,E). In humidity-producing flowers, airflow was through water in a bubbler in this pump assembly that elevated humidity of the air that was fed to the flower head. Less-humid dry flowers were also created, where the pump assembly was the same, but the bubbler was empty. Thus, airflow at the flower head was the same between flower variants but the air reaching the flower head in dry flowers had not had its humidity increased.

A full schematic diagram of an active artificial flower's pump assembly and its installation in the flight arena is given in Fig. 1E. Airflow from a mechanical fish tank air pump (MARINA cool 11135, Hagen) was connected to the bubbler chamber by a 22 cm section airline. The last 7 cm of this 22 cm tube was inserted within the bubbler chamber and the last 2 cm of this section of tube was cut away at a 20 deg angle. This allowed the tube from the pump to be below the water level and allowed surface tension at the end of the pipe to be weaker, promoting movement of bubbles through water when the bubbler was full.

This bubbler chamber was made with an airtight 150 ml Tupperware cylinder (made with either Snac-Pacs food tubes, Wilko, Worksop, UK; or Snack tubes, Smash Nude Food Movers, Mitcham, VIC, Australia). Two holes were drilled into the lid of this and fitted with rubber grommets to match tube diameters. This chamber was either filled with 100 ml of water (that had been allowed to settle at room temperature overnight) in humid flower variants or left empty in dry flower variants. This meant that in humidity-producing flowers, air that had undergone mixing with the water travelled up to the top of the bubbler, while flow of air continued in the same way in dry flowers but without humidity being increased in this air.

A 26 cm section of airline tubing was then connected to a rotameter (Omega FL-3802C, Omega Engineering, Manchester, UK). Only enough tubing of this section to clear the grommet was inserted into the bubbler chamber (3 mm). This meant that this tube was always above the water level and collected humid air, and drier air, at the top of the bubbler. These rotameters regulated airflow using a screw to obstruct airflow. Airflow was set at 2.69 ml s^−1^, controlling the flow of humid or dry air to the artificial flower head. The rotameter output was linked to a 90 cm long section of tubing that entered the flight arena through holes in a wooden bracket installed on the doorways of the foraging arenas. This 90 cm tube would link to the artificial flower itself. Eight active flowers were used at any one time, with four entering the arena from either side through two door brackets (Fig. 1D).

A 25 mm diameter hole was cut into the bottom of a plastic cup (Dart C71-130, Huntingdon, UK), and a 6 mm diameter hole was punched 3 mm from the top on one side, just above the lip on the top. This cup was upturned, and all but the lip was covered with black electrical tape (Fig. 1C). This functioned as the flower stand, holding the artificial flowers upright. The 90 cm tube from the rotameter was fed into the 6 mm hole and up through the 25 mm hole. This base was then weighted using modelling clay, allowing it to stand in place against any elastic tension created by bending the tube.

The head of the artificial flower was made from a specimen jar (Thermo Scientific Sterilin, PS 60 ml, with white plastic lids), where the top 1 cm of the jar (containing the white lid and screw threading of the jar) was cut away. A 0.5 ml Eppendorf tube lid was upturned and stuck down in the centre of the jar lid, to function as the feeding well containing sucrose solution or water; 24 holes were made in the jar lid using a thumbtack pin in lines of 3, each line being at a 45 deg angle from the next, with the first hole at the base of the feeding well and the others separated by 5 mm (Fig. 1A). The screw thread was stuck to thick card using super glue (Precision super glue, Loctite, Hemel Hempstead, UK). Once dry, the joint of this card and the screw thread was covered in glue to ensure as good a seal as possible. A 6 mm diameter hole was then punched through the centre of this card base, and the last 3 mm of the 90 cm tube leading to the rotameter was inserted through it (Fig. 1B) and secured with electrical tape. The flower lid was then screwed tight and the tubing pulled taut so that the flower head would rest on the stand. A small amount of Blue Tac was stuck to the underside of the flower head, to hold it in place against the stand. Artificial flowers thus appeared to bees as the jar lid on top of a trapezoid base (Fig. 1C).

To aid identification of individual flowers by experimenters, in a way bees would not be able to identify, red sticky dots were stuck about the base of the flower stand, and two-digit numbers written on these with black permanent marker (Fig. 1C). These numbers were odd on half of the flowers entering the foraging arena on each side, even on the other half of the flowers (i.e. two of each side's four flowers were even, two were odd). The black on red colours of these numbers would be hard for bumblebees to make out given their visual system (Davies et al., 2013). Additionally, these numbers were two digits, which allowed the initial digit to be an even number in odd number stickers and vice versa. This meant bees were unlikely to recognise a flower based on the number shapes (if they can be seen at all) as even and odd digits were present on all flowers. As the bubblers that contained water could be changed, whether even or odd numbered flowers corresponded with humid or dry flower variants could be changed between experimental days.

### Artificial flower design: passive flowers

Passive flowers created humidity by evaporation of water from components internal to the flower through a permeable lid. In dry, less-humid artificial flower variants, construction was identical but without water being added to the flowers' internal components.

**Fig. 2.**
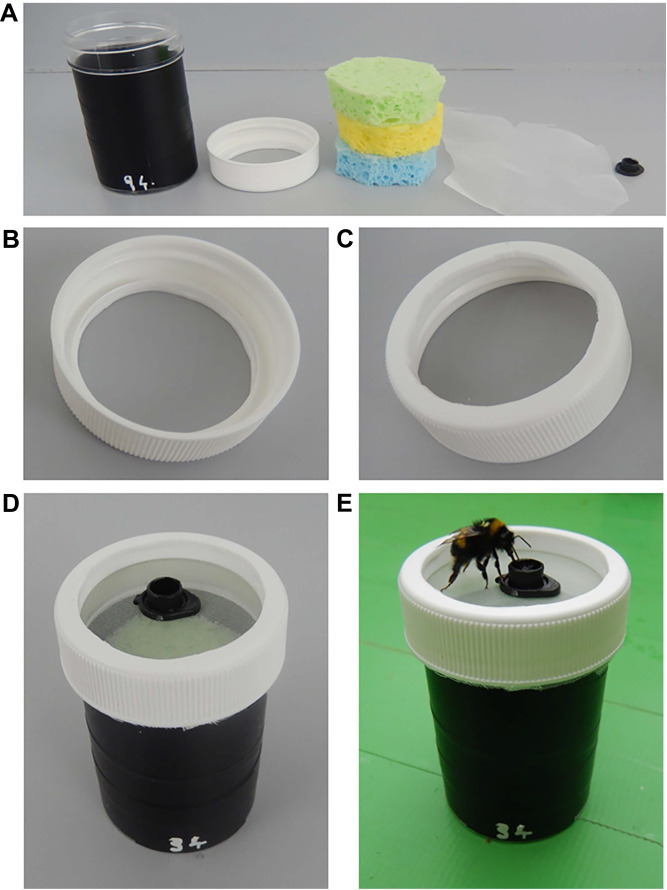
**The passive artificial flowers used in bumblebee experiments.** (A) The artificial flower components, from left to right: the specimen jar; the specimen jar lid; three sponge discs; gauze fabric; and an Eppendorf tube lid. (B) The specimen jar lid showing the cut-away section, leaving the screw assembly, and the lip, from below. (C) The same, but from above. (D) The assembled artificial flower. (E) A bumblebee feeding from the artificial flower.

Passive artificial flowers were built from a specimen jar (Thermo Scientific Sterilin, PS 60 ml, with white plastic lids). The bodies of the jars were covered with black electrical tape to prevent bees visually identifying the artificial flowers by the contents. Flowers were numbered with randomly generated numbers to allow visual identification of humid or dry variant flowers by human experimenters (Fig. 2). Again, these odd and even numbers had several digits, including even and odd digits. This reduced the chance of bees identifying rewards based on the shape of the numbers, as they occurred on both even and odd numbered flowers.

A 35 mm circular hole was cut into the centre of each jar's lid, and the edges were smoothed to remove any excess plastic. This hole removed most of the flat top of the jar but maintained the screw threading assembly of the jar lid (Fig. 2B,C). The top surfaces of the artificial flowers were made with a sheet of fine gauze material (made from cut out segments of TERESIA curtains, IKEA, Leiden, The Netherlands) stretched over the jar aperture and screwed into place using the cut-away lid screw assembly. Any excess gauze visible below the screw lid on all flowers was cut away. This created a gauze top to the artificial flower slightly lower than the plastic rim of the artificial flower (Fig. 2D). This gauze surface was firm enough for the bee to walk upon, would help obscure the jar contents, and was permeable to the evaporation produced by internal components of the artificial flowers (see below). An upturned 0.5 ml Eppendorf tube lid was painted black and placed in the centre of the gauze indentation, functioning as a feeding well during experiments. This lid was not stuck down and could be moved by the bees while feeding; however, it was too heavy for the bees to easily lift and the plastic rim of the artificial flower prevented bees upturning the lid or pushing it off the artificial flower (Fig. 2E).

Three discs of 1 cm thick sponge were placed inside the specimen jars within each artificial flower. These discs (cut from cellulose sponge wipes, Co-op, Manchester, UK) were 40 mm diameter, the width of the specimen jar (Fig. 2A). The top (visible) sponges were all identical green. For humid artificial flowers, these discs were wetted prior to experiments, and at the midpoint of conditioning experiments, as per the protocol in the following section. The evaporation from this wet sponge increased the relative humidity above these artificial flowers. Dry artificial flowers did not have any water added to the sponges.

Each batch of 24 sponge discs was stored in a sealed bag after being cut from sheets until needed. As each flower needed 3 discs and 8 flowers were presented to the bee during trials (see below), all the discs used in one day were from the same sponge batch and stored in the same way. All sponge discs were discarded after a day of use.

### Artificial flower setup

Before preference experiments using active artificial flowers, the pump assembly for four artificial flowers was placed under the flight arena table on both the right and left sides of the arena (as shown for one side in Fig. 1D). This allowed artificial flowers to be placed in the arena through door brackets placed in the doors on that side. On each side, two of the artificial flowers had odd numbering, two even numbering (making eight flowers in total, four odd, four even). The bubbler chambers of either odd or even numbered flowers were filled with 100 ml of water, the other dry, as described above. To ensure a good seal on the Tupperware cylinder and the input and output for the rotameter and grommet seals for the bubbler, all these seals were strengthened with electrical tape. The airflow on all rotameters was then set to 2.69 ml s^−1^ using the rotameter screw seal.

Passive artificial flowers were prepared as follows before each bee's trial in both preference and learning experiments. Sponge discs for dry artificial flower variants were inserted as they were from the bag into the specimen jar before the gauze and flower lid were screwed on. Sponge discs for humid artificial flower variants were submerged until sodden in a pitcher of water that had been allowed to settle at lab temperature overnight, before insertion into the specimen jar and screwing down of the gauze and lid. This was done so that the gauze remained dry, to avoid conflating indicators of which flowers contained wet sponge. If the gauze got wet at any point in the experiment, it was removed and replaced with a fresh dry sheet. Once sponge discs were inserted and tops screwed on, the humidity produced by humid flowers was checked using a handheld hygrometer (Maplin Electronics, Rotherham, UK). If the relative humidity 5 mm above the artificial flowers did not read at least 2% higher than the ambient humidity of the lab using this hygrometer, sponge discs were removed and re-soaked. As humid flowers contained water, insertion of the sponge and evaporation may cause a drop in temperature; therefore, artificial flower temperature was checked before trials began using a thermal camera (FLIR Systems, Inc., Wilsonville, OR, USA). During all thermal imaging, the emissivity parameter value used was 0.95 (Infrared Training Center, 2008) and the reflected temperature of the lab was measured using a tin foil mirror (Harrap et al., 2018) to have a consistent value of 20°C. As the water used for sponge wetting had been allowed to settle at room temperature, humid flower variants and dry flowers rarely differed in temperature enough to elicit a foraging response from bees (where detectability is presumed to occur if the temperature difference is at least 2°C; Heran, 1952). However, if the humid flowers differed in temperature from the dry flowers by more than 1°C, whichever flower variant was warmer would be cooled by placing them on a tray inside a refrigerator at 5°C until the temperature difference between flower variants was below 1°C. If both these humidity and temperature requirements were met, flowers were ready to be presented to bees and experiments could start. During our learning experiments, passive artificial flowers were also re-prepared (as described above) at the end of the foraging bout when the bee crossed the halfway point in terms of visit number (35 visits or more). A foraging bout consisted of the time between a bee leaving the nest to forage in the flight arena and exiting the arena to return to the nest.

### Artificial flower cleaning and maintenance

Both artificial flower types were cleaned regularly throughout the experiments to prevent any conflating scent marks left behind by bee visits (Pearce et al., 2017; Stout and Goulson, 2001). Cleaning occurred at the end of each foraging bout.

When active artificial flowers were cleaned, all flowers were removed from the arena and the tops were wiped with ethanol, with care taken to not apply liquid over the holes. The tubing prevented flowers from being moved to completely different locations during tests, because of the tubes being linked to door brackets, so following cleaning, the door bracket holes by which each artificial flower entered the arena were changed on each side (i.e. a single flower would now enter the arena from a different hole on the same side of the arena). As Tupperware seals, the tightness of the rotameter screw, rotameter input or output seals and grommet seals can weaken under the pressure system of the pump assembly, rotameter airflow was checked after any flower cleaning and the rotameter adjusted. Where necessary, other seals were repaired to maintain a 2.69 ml s^−1^ airflow rate.

When passive artificial flowers were cleaned, there was a risk that the fabric top of the flower retained scent better than plastic parts. Furthermore, returning a passive artificial flower to the arena with an ethanol-wetted top may conflate the humidity differences between flowers under consideration in foraging tests. Consequently, when flowers were removed from the arena for cleaning, the lids and gauze were removed. The plastic parts of the lip were wiped down with ethanol, and a fresh sheet of unused gauze was screwed down onto the flower top with the clean lid. Excess gauze outside of the screw assembly would be cut away as before (see Fig. 2D). This cleaning and replacement of fabric prevented scent marks that might aid reward discrimination from accumulating on the flower tops and allowed artificial flowers to remain consistently dry to the touch of the bees between foraging bouts.

### Artificial floral humidity: sampling

Both humidity-producing and dry variants of both artificial flower types, eight of each active artificial flower variant and 12 of each passive artificial flower variant, were sampled using the robot arm transect-based method described in Harrap et al. (2020a) to evaluate the artificial floral humidity they produce.

This method utilized a Staubli RX 160 robot arm (Pfäffikon, Switzerland). This robot arm carries out paired transects of the flower headspace of (upward-facing) flowers placed on a table in front of it (see Harrap et al., 2020a, for detailed schematics of the robot sampling area setup and transects): first, an ‘*x*-axis transect’ sampling humidity across the horizontal span of flower; second, a ‘*z*-axis transect’ sampling humidity vertically upwards from the flower. Along these transects, the robot samples relative humidity using a DHT-22 humidity probe (Aosong Electronics, Huangpu, China) mounted upon it. Sampling positions of these transects are resolved autonomously by the robot relative to a manually input transect central point, which is a space 5 mm above the flower centre. Simultaneously to humidity sampling by the robot, the background humidity of the sampling area is measured by a background humidity probe on the table flowers are placed upon. The robot carries out these paired transects on each flower presented to it in turn, with a probe calibration step after sampling each flower. In this probe calibration step, the robot moves its humidity probe to the same position as the background humidity probe. Here, the two probes sample the same location, and thus sample an area of equal humidity. Measurements taken in this probe calibration step are used to account for differences in reading between probes (±5% according to the manufacturer's specifications), allowing this source of error to be removed (see Harrap et al., 2020a). The robot then repeats this sequence, sampling each flower in turn (with probe calibrations) 3 more times.

Four artificial flowers were sampled each day (for specific times and dates for each replicate of each variant and type, see Table S3). This means each artificial flower's headspace was sampled 4 times over approximately 21 h within roughly 308 min intervals. Feeding wells of artificial flowers were filled with a 25 μl droplet of water at the start of robot arm sampling, as bees would normally encounter flowers with water or sucrose solution present in the feeding well (see below). Thus, it was necessary to understand what humidity the flowers produce with this water present. All artificial flowers show upwards orientation; therefore, no reorientation of the flowers (as carried out in Harrap et al., 2020a) was necessary. Flowers were placed on the table for sampling. The pump assembly of active artificial flowers was set up under the table within the sampling area of the robot. Flowers were otherwise set up as described previously. Passive humid flowers were not re-wetted at any time after the initial setup during floral humidity sampling by the robot. Setup and activation of the robot for humidity sampling after flowers were prepared was a quick process, taking approximately 10 min.

From these transects we calculated change in humidity relative to the background humidity (ΔRH) across the transects. Once the robot has stopped at a measurement point on each transect, the arm measures humidity ∼100 times in 200 s. These ∼100 measurements taken at each measurement point have been found to have high repeatability of each other (Harrap et al., 2020a). Therefore, the mean ΔRH of each measurement point along the transects was used for analysis. Linear models that allowed differing humidity structures and changes in humidity with replicate transects were fitted to the ΔRH data of each artificial flower variant, as done in Harrap et al. (2020a) for different flower species. Models of different humidity structure that allowed humidity structure and/or intensity to change with replicate transects, or not, were fitted to the *x-* and *z*-axis ΔRH transect data. Models fitted to the *x*-axis data allowed a quadratic, linear or flat relationship, depending on the model, between transect position in the *x*-axis and ΔRH. Models fitted to the *z*-axis data allowed a logarithmic or flat relationship between transect position in the *z*-axis and ΔRH. Throughout all models, artificial flower identity was included as a random factor influencing floral humidity intensity. Further details of the models fitted to humidity data can be seen in the code attached to the datafiles deposited in figshare (https://figshare.com/articles/dataset/Data_from_Bumblebees_can_detect_floral_humidity_/14292320). Best-fitting models for ΔRH across the *x-* and *z*-axis humidity transects of each artificial flower variant were found using AIC. Summary values 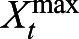 (the point in the *x*-axis transect where humidity difference is greatest) and 
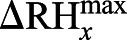
 (the greatest mean humidity difference generated) according to the best-fitting models of each artificial flower variant were calculated. Further details of summary value calculation can be found in Harrap et al. (2020a). This measurement and analysis of floral humidity mirrors those used by Harrap et al. (2020a), allowing direct comparison of floral humidity between artificial and natural flowers.

### Artificial flower humidity: assessment

Floral humidity levels produced by both artificial flower types were comparable to those produced by ‘real’ natural flowers (Harrap et al., 2020a). For the full results of our AIC model comparisons of artificial flower humidity, see Table S1, which contains a summary of best models, Table S2, for full model parameter values, and Table S3, for AIC tables and sampling dates of artificial flower humidity analyses.

**Fig. 3.**
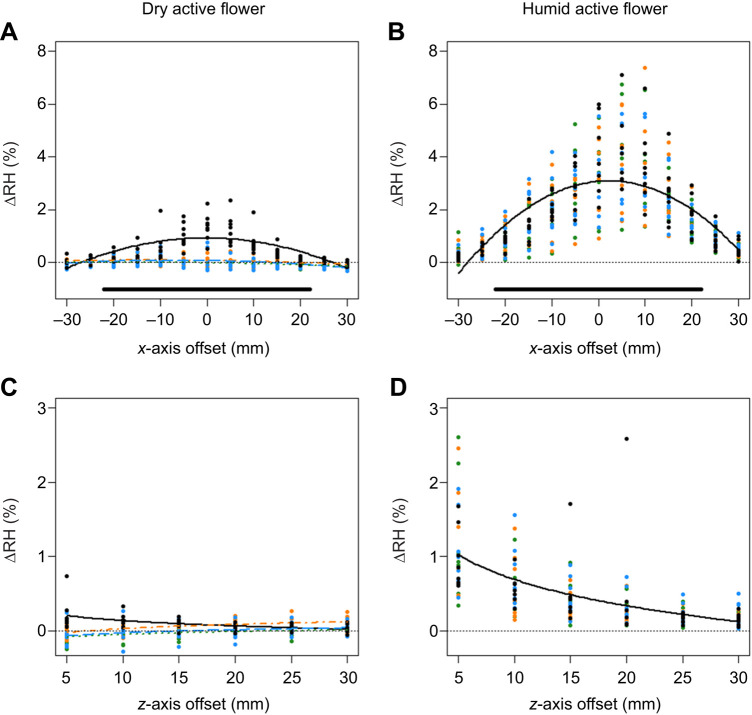
**The difference in humidity relative to the background (ΔRH) for transects of active flowers.** *x-* and *z*-axis transects are given for the dry active flower variant in A and C, respectively, and for the humid active flower variant in B and D, respectively. All axis offsets are relative to the transect central point. The thin dotted line indicates a 0% change in humidity (the background level). Bold lines indicate the mean change in humidity as predicted by the best fitting model for that flower. Colour and dashing of bold lines and points indicate the replicate transect: solid black, first transect; long-dash blue, second transect; dash-dot orange, third transect; dotted green, fourth transect. The solid bar above the *x*-axis transects indicates the diameter of the flower top (44 mm) relative to the *x*-axis. Number of active flowers of each variant sampled, *n*=8.

**Fig. 4.**
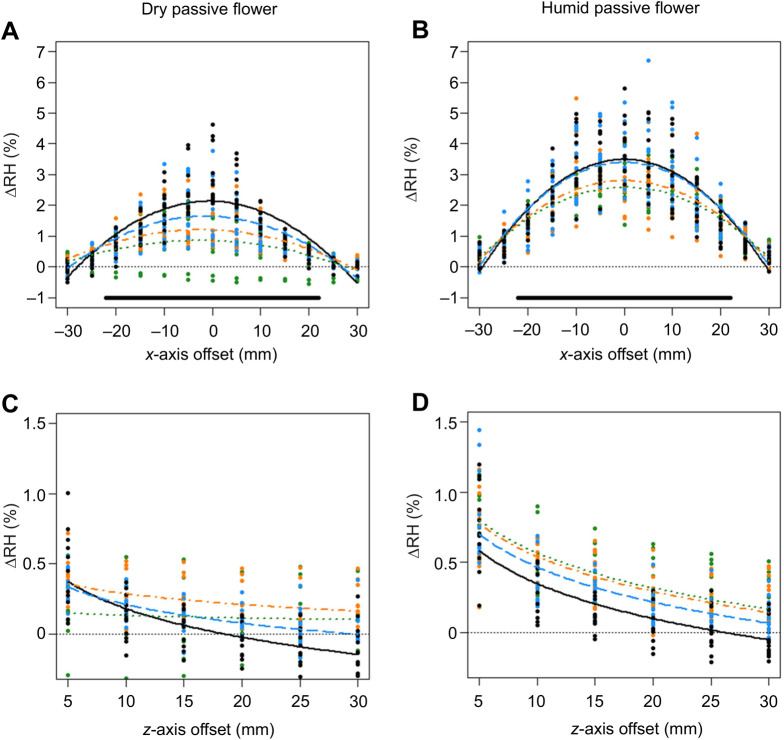
**The difference in humidity relative to the background (ΔRH) for transects of passive flowers.** *x-* and *z*-axis transects are given for the dry passive flower variant in A and C, respectively, and for the humid passive flower variant in B and D, respectively. See Fig. 3 for description of figure details. Number of passive flowers of each variant sampled, *n*=12.

The humidity intensity (
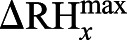
) of active (Fig. 3) and passive (Fig. 4) humid flower variants was 3.08% and 3.49%, respectively. This humidity intensity is comparable to floral humidity produced by real flower species with 
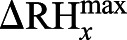
 values greater than 3%, the larger floral humidity intensities observed in nature, such as: *Calystegia sylvatica* (3.71%), *Eschscholzia californica* (3.24%), *Taraxacum* agg. (3.35%) or *Ranunculus acris* (3.41%). Some humidity came from water droplets in the well of the artificial flowers, explaining how humidity was still produced by dry flower variants. The humidity produced by the feeding well was likely to be lower in the dry active flowers than in the dry passive flowers because of the effect of the airflow in active flowers dispersing water vapour (Figs 3 and 4). However, this production of smaller amounts of humidity in the dry variants of both active and passive artificial flowers was both lower than that of humid variants and similar to that produced by real flowers (Harrap et al., 2020a). Active dry flowers produced humidity differences 
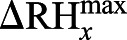
 of 0.92% similar to *Convolvulus sabatius* (0.87%), *Cyanus segetum* (1.10%) and *Linum usitatissium* (0.8%)*.* Passive dry flowers produced humidity differences of 2.13%, which is similar to flowers producing moderate amounts of floral humidity such as *Leucanthemum vulgare* (1.79%) and *Achillea millefolium* (1.73%). The differences in relative humidity intensity between humid and dry flower variants was 2.16% in active flowers and 1.36% in passive flowers. These differences in humidity intensity were similar to those observed between different flowers (Harrap et al., 2020a), which means that the floral humidity levels that the experimental bees were exposed to were within the bounds of differences they might experience when foraging on natural flowers.

Spatially, the humidity was distributed in similar ways to that found in flowers (Harrap et al., 2020a). Peak values were measured in the central area when probing in the horizontal plane across the upward-facing surface (*x*-axis, Fig. 3A,B, Fig. 4A,B), and declined when moving away upwards from the surface (*z*-axis, Fig. 3C,D, Fig. 4C,D).

Humid variants of both flower types produced elevated humidity in the first replicate transect (Figs 3 and 4), instead of humidity increasing over time. This indicates that artificial flowers do not need long for humidity cues to establish themselves, producing elevated humidity shortly after preparation and placement for sampling. The humidity produced by both variants of active flowers remained largely stable throughout the ∼21 h sampling period, during which the humidity of their headspace was sampled 4 times (Fig. 3). The only change observed during this period was a drop in humidity of the dry active flowers after the initial transect (Fig. 3A), which was affected more strongly by the evaporation of the water in the feeding well during the initial transect. As dry active flowers were regularly refilled throughout bee experiments after being emptied (see below), it is likely that the humidity differences were maintained at levels shown in the initial transect. So, the mean difference in humidity intensity (in terms of 
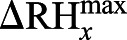
) between dry and humid active flower variants remained ∼2.16% during experiments. The passive flowers were less stable, with the floral humidity regularly dropping with replicate transects in the dry flower variant (Fig. 4A) and dropping after the second replicate transect and again after the third in the humid flower variant (Fig. 4B). This was caused by the drying out of wet sponge components as well as the evaporation of water from the feeding well. During the experiments with bees, the feeding wells of passive flowers were refilled and, where appropriate, sponge components re-wetted. As the passive humid flowers show stable average humidity intensities for the first and second transect replicates, this means that the initial peak in humidity lasted for at least 10 h before drying out affected humidity intensity. Preference and learning trials rarely took this long, so it is unlikely that the humidity would drop much below the initial intensities in the time allowed between re-wetting. Thus, the mean difference in humidity intensity (in terms of 
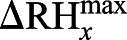
) between dry and humid passive flower variants remained at ∼1.36% within the time scales of our experiments.

### Bee trials

Two kinds of experiments were carried out on captive bumblebees. Firstly, preference experiments (e.g. Dyer et al., 2006; Lehrer et al., 1995; von Arx et al., 2012) were carried out using both artificial flower types. Secondly, differential conditioning techniques (e.g. Clarke et al., 2013; Dyer and Chittka, 2004; Harrap et al., 2017; Lawson et al., 2018) were carried out with passive artificial flowers only. This was because of limits to how much and how quickly active artificial flowers could be moved about the arena due to the piping. Both preference and learning (differential conditioning) experiments tested the capacity of bees to detect and respond to floral humidity differences. Additionally, preference experiments investigated how differences in floral humidity between flowers may influence flower choice of naive bees with no previous experience of floral humidity, in the absence of any other differences between flowers that would significantly affect foraging behaviours. Learning experiments (differential conditioning) investigated whether bees can associate differences in floral humidity with corresponding differences in rewards and use this to inform foraging choices. Therefore, the two experiments together assessed different ways that floral humidity might influence the foraging behaviours of bees. Individual bees were not reused between experiments: an individual bee would only take part in one experiment (preference or conditioning) as part of a single test group (see below) or on a single type of artificial flower (active or passive). Experimental trials were not time controlled or limited, and the length of experiments was measured in terms of the number of flower visits made by bees. Such procedures are typical for bee foraging trials (Clarke et al., 2013; Dyer and Chittka, 2004; Harrap et al., 2017, 2019; Lawson et al., 2017a, 2018); as bees will return to nests during foraging, time is not as appropriate an indicator of flower exposure as the number of visits made. However, individual trials all took place between the hours of 09:00 h and 19:00 h, and individual bees completed trials on the same day that they started. If a bee failed to complete the experimental trial after being presented with flowers for testing on the day it started, it would not be reused, and any observations associated with it would be excluded.

### Preference experiments

Two different bumblebee nests were used in the passive flower tests. Bees used in the active flower tests came from four different nests, which included the two nests used in the passive flower tests. During preference tests, bees were presented with eight artificial flowers of the type assigned to them, placed randomly about the foraging arena floor. Four of these were the humid flower variant, and the other four were the dry flower variant. All artificial flowers were rewarding, containing a 25 μl droplet of 30% sucrose solution within their feeding wells.

Individual marked bees were released into the arena alone (that is, one bee at a given time was allowed in the arena to undergo the preference experiment), and bees were allowed to forage freely on the presented artificial flowers. Bees were free to return to the nest at all times. As the foraging bees were marked, upon return from the nest they could be identified and released back into the arena for the next foraging bout. Typically, upon encountering artificial flowers, a foraging bee slows down flight and makes contact with the tops of artificial flowers with its feet, preparing for landing. After landing, it either proceeds to extend its proboscis into the feeding well (feeding), or departs without feeding. We monitored which variants of flowers (humid or dry) bees landed upon and whether they extended the proboscis into the feeding well (‘fed’). If the bee touched the top of the flower with its feet (‘contact’ in the above description) but departed shortly after, we considered it to be a landing without proboscis extension (as done by Harrap et al., 2017, 2019), as the bee had entered the flower headspace. No instances were observed to suggest that bees could not control their flight within the arena or collided with flowers, and many studies have previously used the same type of flight arena to investigate floral responses in bees.

After a bee had departed from a flower, the flower was refilled if the bee had emptied it and moved. Bees are highly capable of learning the locations of rewarding flowers (Burns and Thomson, 2006; Robert et al., 2017), particularly within the small area of a flight arena. It would be possible for bees to learn to return to the locations where rewards had been found previously. Therefore, as in previous studies (Harrap et al., 2017), we carefully changed flower position after a bee departed from a flower and whilst it was in another part of the arena. For the passive flowers this involved taking the flower out of the arena and placing it back in a different position. With active flowers, the ability to move the flowers was limited by their pipes and the flowers' current arena entry points. Consequently, active artificial flowers were not taken out of the arena but were instead moved to a different point. In the rare instances where a bee left a flower and then revisited it before it could be moved, these revisits were not counted. When the bee returned to the nest, all the flowers were removed from the arena, cleaned and returned to the arena in a new position as described previously. Movement of flowers could potentially interfere with humidity cues. However, as flower variants were treated in the same way, humid flower variants would still produce more humidity relative to dry variants. Furthermore, flowers did not need to be left long to establish humidity cues, as seen in robot sampling (Figs 3 and 4), so this effect was assumed to be small.

For each test bee, this cycle of moving flowers continued until the bee had made 20 flower landings. This was normally achieved in 4.38±0.44 (mean±s.e.m.) foraging bouts (a ‘foraging bout’ being the time between departure and return to the nest) with an average of 5.06±0.41 visits per bout for bees presented with active flowers and 3.00±0.34 bouts with an average of 7.40±0.67 visits per bout for those presented with passive flowers. Despite the lack of a pre-training phase, the naive bees foraged readily on artificial flowers. Of bees presented with active artificial flowers for preference tests, 66.7% (18 of 27 bees) at least began to visit them, and 88.9% of these (16 of 18 bees) completed the preference test (reaching 20 flower visits) and were included in the analysis. Similarly, of bees presented with passive artificial flowers for preference tests, 65.5% (19 of 29 bees) at least began to visit them, and 84.2% of these (16 of 19 bees) completed this preference test and were included in the analysis. Bees that failed to at least begin visiting test flowers will include foragers unable to manipulate these artificial flowers, but also erroneously marked non-forager bees. Bees that did not complete the test may reflect the colony becoming satiated during foraging, leading to the bee not returning to the arena to forage, or a loss of motivation by the bee as a result of fatigue. Sixteen bees completed the preference experiment on each type of artificial flower (32 bees in total).

For each flower visit made, we determined whether the bee demonstrated a response in favour of elevated floral humidity or not. As bees may land upon flowers that they reject by departing from the flower without attempting to drink, it was important to consider both the identity of flowers bees landed upon and whether bees showed proboscis extension upon landing when classifying whether behaviours are indicatory of a preference. Flower landings recorded in favour of floral humidity were either landing on a humid flower and extending the proboscis into the feeding well (indicating choice of flowers with elevated humidity), or landing on a dry flower and leaving without extending the proboscis into the feeding well (a rejection of low floral humidity). Responses that were recorded as not in favour of elevated floral humidity were either landing on a dry flower and extending the proboscis into the feeding well (indicating choice of flowers with lower humidity), or landing on a humid flower and leaving without extending the proboscis into the feeding well (a rejection of flowers with elevated humidity). This classification of flower landings as indicatory of a preference toward floral cues is identical to those used in previous studies with preference trials with bees (Dyer et al., 2006; Harrap et al., 2019; Lawson et al., 2018), and other animal studies. It is possible that bees landed on flowers for other reasons than seeking reward, such as grooming, that may result in rejections not indicatory of humidity preferences. However, such flower landings were probably rare; we observed that landings followed by grooming also took place on the arena floor, and would be as likely to occur on either flower variant.

For each bee, a ‘humidity response rate’ was calculated; this was the proportion of the 20 flower landings in the preference trials that demonstrated a response in favour of elevated humidity. The humidity response rate is equivalent to other identical metrics applied to other cues in previous studies (Harrap et al., 2019), including measures like ‘percentage preference’ (Dyer et al., 2006). If bees were foraging randomly, we would expect a humidity response rate of 0.5, as humid and dry flowers occur equally, while a humidity response rate above or below 0.5 indicates the bee favours humid flowers or dry flowers, respectively. By analysing whether humidity response rates differed from 0.5, we assessed whether bees showed a preference for or against elevated humidity. The humidity response rate data were bounded between 0 and 1, and so were arcsine square-root transformed to fit test assumptions. We used a two-tailed one-sample Wilcoxon signed-rank test to test whether the median value of the transformed humidity response rate differed from that expected from random choice (a 0.5 humidity response rate, 0.79 once arcsine square-root transformed), using R 3.6.3 (http://www.R-project.org/).

Temperature differences between humid or dry flower variants might occur as a result of differing evaporative water loss between humid and dry passive artificial flowers, or differing transfer of heat from the action of mechanical components within humid and dry active artificial flowers. Air temperature can influence the amount of water vapour indicated by a given relative humidity value, a rise of 10°C approximately doubling the vapour indicated by relative humidity (Tichy and Kallina, 2014), so a large difference between floral temperatures of flower variants may influence flower headspace temperature, conflating humidity cues. Bees can also respond to floral temperature, showing preferences and learning (Dyer et al., 2006; Harrap et al., 2020b, 2017; Whitney et al., 2008). Differences in floral temperature above a level that would induce a foraging response in bees (presumed to be a temperature difference between flowers of at least 2°C; Heran, 1952) may also conflate bee responses. Furthermore, temperature and humidity perception are linked in insects (Budelli et al., 2019; Enjin, 2017; Enjin et al., 2016; Frank et al., 2017; Hernandez-Nunez et al., 2020 preprint). So, temperature differences may interact with humidity perception, influencing pollinator responses, perhaps leading to enhanced responses or contextual responses (e.g. preference for humidity that is dependent on flower temperature). For these reasons, flower temperature differences of artificial flowers were monitored alongside the preference experiment, using a thermal camera (FLIR E60bx, FLIR Systems), to see whether the flowers developed a floral temperature difference of a level that could alter the foraging behaviour of the bees. This was done at the start of foraging or after flower cleaning at the end of foraging bouts, by randomly selecting one humid and one dry artificial flower and measuring the temperature of the flower top. This procedure for checking flower temperature did assume that the temperatures of this pair of flowers were representative of each variant at the time of sampling. The emissivity parameter value used was 0.95, an accepted value for plastics (Harrap et al., 2018), and reflected temperature used was a consistent value of 20°C.

### Learning experiments

In the learning experiments, individual marked bees were allowed into the arena alone and were presented with eight passive artificial flowers placed randomly about the flight arena – four rewarding and four non-rewarding – with humidity production by these flowers assigned as per the bee's test group (described below). Bees were allowed to forage freely on these artificial flowers, and again allowed to return to the nest as required. As individuals were marked, they could be identified and allowed to return to the arena from the nest to resume foraging in the experiment. We monitored both whether the bee landed on rewarding or non-rewarding artificial flowers (landing defined as described in the preference test) and whether the bee extended its proboscis into the feeding well or left without doing so at each landing.

Before a trial began, bees were assigned to one of three test groups: (i) ‘humid reward’ group, where the humid passive flowers were rewarding and the dry passive flowers non-rewarding; (ii) ‘dry reward’ group, where the rewarding flowers were dry passive flowers, and humid passive flowers were non-rewarding; (iii) ‘control’ group, where none of the flowers produced humidity (i.e. both rewarding and non-rewarding were dry passive flowers), meaning that flowers only differed in their rewards. Excluding differences in cues associated with rewarding and non-rewarding flowers (determined by which flower variants were rewarding or non-rewarding), bees in each of the three test groups were otherwise treated with exactly the same experimental procedures. Rewarding flowers had a 25 μl droplet of 30% sucrose solution within their feeding wells and non-rewarding flowers contained a 25 μl droplet of water. Four different bumblebee nests were used in this experiment; none of these nests were used in preference experiments.

The control group was required for checking to what extent bees could use any miscellaneous cues other than humidity or variables present in the experimental setup to solve the task. Such miscellaneous cues may include the shapes of the numbers on the side of the flowers (although effort was made to reduce this possibility, see details of artificial flower construction), or small differences in the shape of the cut-away components of the flowers' lids (see details of artificial flower construction) that may influence the appearance or tactile properties of individual flowers. The arrangement of flowers in the arena, such as incidental clustering of rewarding or non-rewarding flowers, or some combinations of environmental cues within the arena may also facilitate learning in some manner. The appearance of flowers in novel positions with repositioning during trials (see below) might also indicate reward presence or absence, dependent on the bees' previous visits. These miscellaneous variables might give rise to a basic capacity of bees to find individual rewarding flowers, independently of humidity differences, within the specific elements of our setup. By comparing bee responses between the control group and other test groups, we were able to assess the extent to which humidity differences between flowers alone influenced bee foraging. This is reflected in our analysis, which compares bee responses between test groups.

Bees were observed for 70 flower visits, which is well beyond the number of visits needed for bees to learn a salient cue, and sufficient to demonstrate such learning by a consistent change in foraging choices (e.g. Clarke et al., 2013; Harrap et al., 2017; Lawson et al., 2018). Of bees presented with flowers for the learning trial, 62.8% (59 of 94 bees) at least began to visit them, and 76.3% of these (45 of 59) completed the test (reaching 70 flower visits) and were included in our analysis; 15 bees completed this learning trial in each test group (45 bees in total). These bees achieved 70 visits in, on average, 5.13±0.31 foraging bouts (mean±s.e.m.) with 13.78±0.56 landings in each bout. The longer foraging bout lengths here compared with the preference trials is likely to reflect the experimental setup differences between the learning experiments (where half the flowers presented are non-rewarding) and the preference trial (where all flowers are rewarding). Upon proboscis extension into feeding wells of non-rewarding flowers, bees usually consumed only small amounts of the water provided before they departed from the flower, while when feeding from a rewarding flower, bees consumed most of the sucrose solution provided. If a bee associated floral cues with a lack of reward, it would also become less likely to extend its proboscis and attempt to feed during a visit to flowers that it recognised as non-rewarding. The foraging bouts in the learning trial were therefore more likely to include visits where the bee either did not feed or fed less. Consequently, more visits would be needed to fill the bee to the point at which it became motivated to return to the nest.

After a bee departed from a flower and flew off into another part of the arena, that flower was carefully removed from the arena through the side openings and refilled if required, with sucrose or water as appropriate, before being placed back at a different location. This reduced the chance of bees associating particular spatial locations with the reward. If a bee suddenly revisited the flower before it could be moved, then these revisits were not counted. When the bee returned to the nest, all the flowers were removed from the arena, cleaned and returned to the arena as described previously. The moving of flowers during the experimental trial may slightly disrupt humidity cues, meaning cues presented to bees may be slightly lower humidity than that found during robot sampling. However, given the humidity cues appeared to establish themselves quickly (as discussed above), this effect was likely to be small and even in the event of such effects, humid flowers would be likely to still maintain elevated humidity relative to dry flowers.

For each flower visit, we determined whether the bee's foraging decisions were ‘correct’ or ‘incorrect’ in response to the floral rewards presented. As bees can land on and inspect flowers before rejecting them, it was important (as in the preference test) to consider both the landing decisions and the subsequent proboscis extension decisions when determining whether the bee performed a correct foraging action at each landing. Classification of each visit as correct or incorrect was done using the same criteria described in our previous studies (Clarke et al., 2013; Foster et al., 2014; Harrap et al., 2017, 2019; Whitney et al., 2008, 2016). A bee was recorded as making a correct decision if she landed on a rewarding flower and extended her proboscis into the feeding well, or if she did not extend her proboscis into the feeding well after landing on a non-rewarding flower. Correspondingly, a bee was recorded as making an incorrect decision if she landed on a non-rewarding flower and extended her proboscis into the feeding well, or did not extend her proboscis into the feeding well when she landed on a rewarding flower. As in the preference tests, bees may have landed upon flowers for reasons other than foraging that may result in rejections not indicatory of foraging decisions. However, much like in the preference trials (see above), it is likely that bees will perform such landings rarely and as frequently on rewarding or non-rewarding flowers.

The success rate, i.e. the proportion of correct visits made over the previous 10 visits, was calculated at 10 visit intervals (10 visits, 20, 30… etc.) for each bee. Success rate is identical to measures used in other studies (Harrap et al., 2017, 2019), and equivalent metrics that record incidence or proportions of correct decisions (Dyer and Chittka, 2004; Foster et al., 2014; Whitney et al., 2016, 2009b). Success rate indicates foraging success of bees by indicating accurate foraging decisions, correctly visiting rewarding flowers and limiting expenditure on non-rewarding flowers. If a bee is foraging randomly, we would expect a success rate of 0.5; as half the flowers are rewarding and half non-rewarding, the bee is equally likely to make the correct or incorrect decision, while higher success rates indicate bees perform more correct actions. Improved success rates with experience of flowers (increased number of visits made by the bee) indicates that bees can learn to identify rewarding flowers, necessitating its calculation at 10 visit intervals. As it was bounded between 0 and 1, the success rate data were arcsine square-root transformed to fit test assumptions. Generalised linear models were fitted to these data using R, and AIC model simplification techniques (Richards, 2008) were used to analyse the effects that experience of the flowers (number of visits made) and test group (the presence of floral humidity differences between rewarding and non-rewarding flowers) had on bumblebee foraging success (success rate). The models used for analysis and AIC model simplification procedure are described in detail in the following section.

### Statistical models and simplification procedure for bee learning experiments

The following represents the initial model of bee learning before any simplification was applied; these models are similar to those employed in Harrap et al. (2017, 2020b):(1)
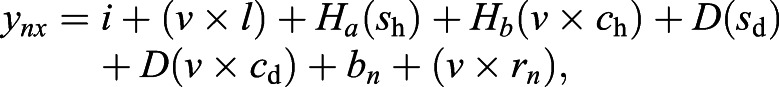
where *y_nx_* is the arcsine square-root success rate of bee *n* over the previous 10 visits to the artificial flowers, at *x* flower visits, *v* is related to the number of flower visits the bee has made to the artificial flowers, *x*, by the following:(2)
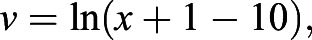
and the data for *y* are calculated in blocks of 10 visits (i.e. at 10, 20, 30, 40, 50, 60 and 70 visits). The transformation shown in Eqn 2 allows the model to show a logarithmic relationship and the model intercept to be the first calculated success rate (that achieved by bees at 10 visits). Parameter *i* is the initial arcsine square-root success rate, the model intercept, for bees in the control group when *x*=10. Parameter *l* dictates the change in arcsine square-root success rate with increased *x* in the control group, thus *l* is effectively the learning speed parameter and allows the bee's experience to effect success rate. *D*, *H_a_* and *H_b_* are Boolean parameters which allow the model to alter *y* depending on which test groups the bee is in. *H_a_* and *H_b_* are identical (the use of different parameters to describe the same Boolean is for ease of reference during model simplification, see below) and indicate whether bees are presented with rewarding and non-rewarding flowers that differ in humidity, where:(3)
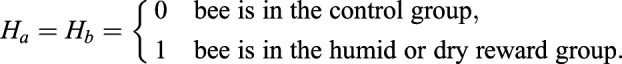


Boolean *D* indicates whether the bee is in the dry reward test group, where:(4)
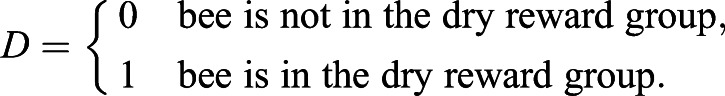
Parameters *s*_h_ and *c*_h_ are the change in initial arcsine square-root success rate and learning speed, respectively, for bees that are in either the dry or humid reward test groups, relative to *i* and *l*. Parameters *s*_d_ and *c*_d_ are the change in initial arcsine square-root success rate and learning speed, respectively, for bees that are in the dry reward test group, relative to *s*_h_ and *c*_h_. In this way, when parameters *s*_d_ and *c*_d_ have non-zero values, *s*_d_ and *c*_d_ represent the differences in initial success and learning between bees in the humid and dry reward test groups, while *s*_h_ and *c*_h_ describe the differences in initial success and learning between bees in the humid reward and control groups. If parameters *s*_d_ and *c*_d_ equal zero, initial success and learning of bees in the dry reward group would be the same as those in the humid reward group, being determined solely by *s*_h_ and *c*_h_. In this scenario, *s*_h_ and *c*_h_ would describe a common initial success and learning, respectively, of bees presented with flowers that differ in floral humidity. Variation between individual bees was included in our model as a random factor. *b_n_* and *r_n_* represent the change in initial arcsine square-root success rate and learning speed, for bee number *n*. In the model described in Eqn 1 parameters *I*, *l*, *s*_d_, *s*_h_, *c*_d_, *c*_h_, *b_n_* and *r_n_* are parameters to be estimated.

The model simplification procedure involved paired comparisons between the standing ‘best model’, beginning with the full model described in Eqn 1, with a simpler model. Simpler models were constructed from the standing best model but with further parameters removed (effectively forcing the relevant parameters to equal zero) and tested against the standing best model in the sequence described below. Should the simpler model have a lower AIC or be comparable to the standing best fitting model based on AIC, as laid out by Richards (2008), this simpler model would become the standing best model for the next comparison. If removal of a parameter led to a significant increase in AIC, again as laid out by Richards (2008), the standing best (more complex) model would remain the best for the next comparison.

Initially, the effects of random factors were compared. A model without *r_n_* was compared with the initial model (Eqn 1). This allowed testing of whether individual bees differed only in intercepts or intercepts and learning speed (as in the initial model). Next, differences in learning speed between the dry and humid reward test groups were tested by removing parameter *c*_d_. Then, differences in the initial success rate (model intercept differences) between dry and humid reward test groups were tested for by removal of *s*_d_.

If these AIC comparisons indicated no differences between the dry and humid reward test groups, that is both *s*_d_ and *c*_d_ should not be retained in the standing best model, the difference in the learning speed between bees in the control group and the common learning speed of bees in the dry and humid reward test groups was tested for by removing *c*_h_. Then, a difference in the initial success rate (model intercept) between bees in the control group and the common initial success rate of bees in the dry and humid reward groups was tested for by removing *s*_h_. Lastly, the extent to which background learning occurs, i.e. whether bees in the control group (or groups that learn at the same speed should *c*_h_ be removed from the standing best model), was tested by removal of parameter *l*.

If previous AIC comparisons indicated any differences between the dry and humid reward test groups, i.e. that one or both of *s*_d_ and *c*_d_ should be retained in the standing best model, the between test group comparisons described by the model were adjusted to allow comparisons of any separate test group responses with those of the control group. This adjustment of the model's comparisons was necessary if dry and humid reward groups were found to differ in intercept or learning, as these groups could differ from each other but not the control group; for example, if a change in intercept or learning speed occurred in only one test group. If the previous AIC comparisons indicated *s*_d_ should be retained in the standing best model, *H_a_* would be replaced with *H_c_*, where:(5)
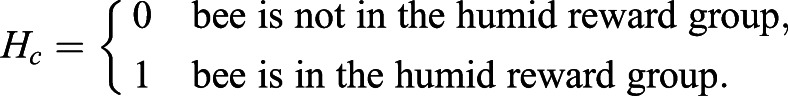
Likewise, should *c*_d_ be retained in the standing best model, *H_b_* would be replaced with *H_c_*. Such adjustments result in an equivalent model to the standing best model but where parameters *s*_d_ or *c*_d_ (when retained in the model) now describe changes in initial success and learning of bees in the dry reward group relative to *i* and *l*, the responses of the control group. Once the model comparisons are adjusted, the presence of differences in learning and then initial success between the control group and the dry reward group would be tested by (again) removing *c*_d_ and then removing *s*_d_ (if retained in the model). Differences in learning and then initial success between the control group and the humid reward group (or any shared responses of both groups depending on previous comparisons) were then tested by removing *c*_h_ and then *s*_h_. Lastly, as before, the presence of learning independently of experimental test group was tested by removal of parameter *l*.

An annotated copy of the code used in this analysis is available attached to the datafiles deposited in figshare (https://figshare.com/articles/dataset/Data_from_Bumblebees_can_detect_floral_humidity_/14292320).

## RESULTS

### Artificial flower temperature differences

Artificial flower temperature differences, as measured during the preference experiments, were negligibly small (data for floral temperature measurements are available from figshare: https://figshare.com/articles/dataset/Data_from_Bumblebees_can_detect_floral_humidity_/14292320). Dry passive flowers had a temperature that was 0.31±0.03°C (mean±s.e.m.) higher than that in humid passive flowers throughout the experiment, evaporation of wet internal components in humid passive flowers cooling them slightly. In active artificial flowers, flowers of the humid and dry variants differed even less in temperature. Dry active flowers were 0.03±0.03°C colder than humid active flowers, the presence of water in humid flower pump assemblies slightly increasing heat transfer from active flower pumps. Measured differences between dry and humid flower variants (dry flower variant temperature minus humid flower variant temperature) ranged from −0.2 to 0.9°C in passive flowers and −0.5 to 0.5°C in active flowers. These differences in temperature were below temperature differences that elicit a foraging response by bumblebees (Heran, 1952) and are thus unlikely to elicit a foraging response by bumblebees.

### Bee trials

**Fig. 5.**
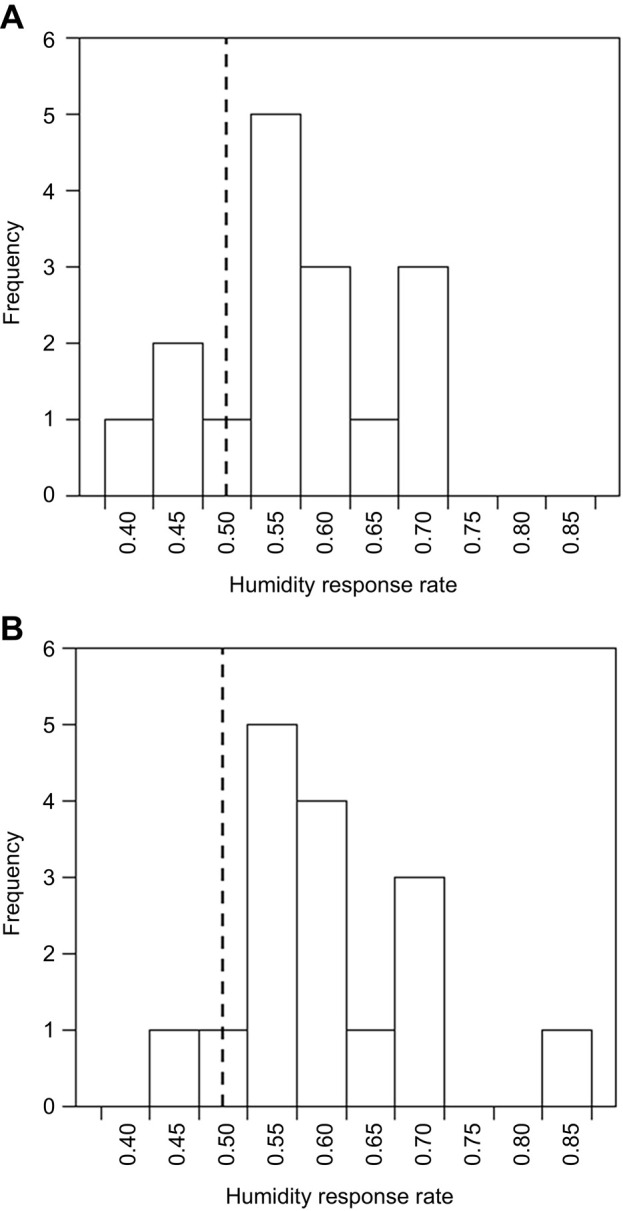
**Histograms showing the responses of bumblebees to rewarding passive and active flowers in the preference experiments.** (A) Passive flowers; (B) active flowers. Bars represent the frequency of bees (*n*=16 bees in each trial) that over 20 flower landings achieved each humidity response rate (the proportion of landings in which the response of the bee was in favour of the elevated floral humidity of humid flowers, as opposed to the lower floral humidity of dry flowers). Dashed vertical line indicates the expected humidity response rate for randomly foraging bees (0.5).

In preference experiments, bumblebees showed a higher spontaneous preference for humid flowers when they were allowed to freely choose between four humid and four dry flowers providing sucrose solution (Fig. 5). The median humidity response rates differed significantly from that expected from random foraging (0.5), in tests with both passive flowers (Wilcoxon test, *W=*109, *n=*16, *P=*0.006) and active flowers (Wilcoxon test, *W*=119, *n=*16, *P=*0.001). The median bee humidity response rates in both preference tests were greater than 0.5 (Fig. 5), indicating bees on average showed a preference for elevated floral humidity.

In the learning experiment, bumblebees were presented with passive flowers differing in rewards, providing either sucrose solution or water in feeding wells. The humidity cues corresponding with rewards varied dependent on the three test groups bees were assigned to (see above). The relationship between foraging success (measured by success rate, reflecting incidence of probing the feeding wells of rewarding flowers, or not probing it on non-rewarding flowers) and the experience bees had of the flowers (number of flower visits the bees made) was compared between the three test groups to evaluate the capacity of bumblebees to learn to identify rewarding flowers based on humidity differences.

**Fig. 6.**
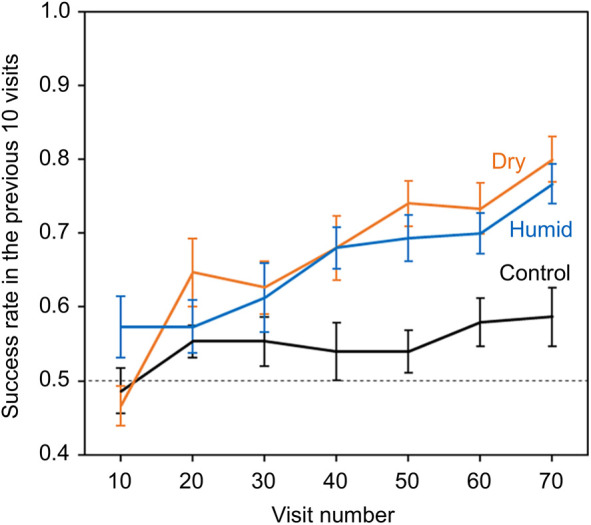
**The relationship between bees' foraging success and experience of passive artificial flowers (flower visits the bee made).** Dotted line indicates the 50% success level. Solid lines indicate the mean foraging success of bees in the previous 10 visits. Error bars represent ±s.e.m. Colour and label of solid lines and error bars correspond with test group: black, control group; orange, dry reward group; blue, humid reward group. Number of bees in each test group, *n*=15.

The presence of floral humidity differences between rewarding and non-rewarding flowers influenced the ability of bumblebees to learn to identify rewarding flowers and the foraging success achieved by bees (Fig. 6). Models that allowed individual bees to have different intercepts as well as different learning speeds independent of their test group (models which had random slopes and intercepts) were not a better fit than those that only allowed individual variation in intercepts (AIC: random slopes and intercepts −291.87 versus random intercepts only −294.32, ΔAIC=2.45, Δdeviance=1.55, d.f.=2, *P=*0.461). Models that allowed the dry reward test group to differ from the humid reward group in learning speed did not perform sufficiently better in terms of AIC (see Richards, 2008) than models where these two groups showed a common learning response (AIC: different learning speeds −294.32 versus common learning speeds −290.47, ΔAIC=3.85), but were of better fit (Δdeviance=5.86, d.f.=1, *P=*0.016). Similarly, models that allowed the dry reward test group to differ from the humid reward group in model intercept were not better than those where these groups showed a common intercept (AIC: different intercepts −290.47 versus common intercepts −291.76, ΔAIC=1.29, Δdeviance=0.707, d.f.=1, *P*=0.401). The results of these AIC comparisons indicate bees in the dry and humid rewards groups did not differ in their responses; that is, groups where floral humidity differed between rewarding and non-rewarding flowers show a common initial success rate and common changes in success rate with experience.

Bees in the control group, where artificial flowers produced no humidity differences, began the experiment with a success rate around 0.5, and improved only slightly, maintaining a success rate just above this over the rest of the experiment. In the dry reward and humid reward test groups, where floral humidity differed between rewarding and non-rewarding flowers, bees began foraging at a success rate comparable to that of the control group, but success improved as bees made more flower visits, with these groups achieving a greater level of success than those in the control group (Fig. 6). Consequently, models that allowed (the common) learning speed of bees in the dry and humid reward groups to differ from that of bees in the control group had a lower AIC and better fit (AIC: different learning speeds −291.76 versus equal learning speeds *−*283.59, ΔAIC=8.17, Δdeviance=10.17, d.f.=1, *P*=0.001). However, models that allowed the (common) initial success of bees in the dry and humid rewards test groups to differ from that of the control group did not perform better in terms of AIC and were of poorer fit (AIC: different intercepts −291.76 versus equal intercepts −293.75, ΔAIC=1.99, Δdeviance=0.013, d.f.=1, *P*=0.910). Models that allowed the success of control group bees to change with experience were not sufficiently better than those that allowed no change in success for control group bees (AIC: experience effects −293.75 versus no experience effects −288.67, ΔAIC=5.08), although these models were a better fit (Δdeviance=7.08, d.f.=1, *P*=0.007). This indicates that the presence of floral humidity differences between rewarding and non-rewarding flowers improved bumblebee foraging success and learning of the identity of rewarding flowers with experience, regardless of whether rewarding flowers produced higher or lower floral humidity intensities.

## DISCUSSION

By experimentally varying the levels of floral humidity in artificial flowers within a range that is biologically relevant, we show here that bumblebees are able to detect and utilise humidity differences in a flower foraging context. In an all-rewarding array with artificial flowers that offered low and high floral humidity cues, bumblebees showed an unlearned preference for flowers with elevated floral humidity (Fig. 5). Our finding aligns well with previous observations in the hawkmoth *H. lineata* (von Arx et al., 2012) and field observations of alpine fly pollinators in India (Nordström et al., 2017), which suggested insect pollinator preferences for flowers with higher floral humidity. When rewarding and non-rewarding flowers differed in humidity cues, bumblebees showed enhanced foraging success with experience compared with bees in the control group (Fig. 6), where rewarding and non-rewarding flowers did not differ in humidity. This indicates that bumblebees can associate floral humidity with the presence or absence of a nectar reward. Therefore, floral humidity differences enhanced learning of rewarding flowers. When rewarding and non-rewarding flowers differed in floral humidity, bees began foraging comparably to the control group but with experience learned to favour visits to the rewarding flower type, whether rewarding flowers were producing higher or lower levels of floral humidity. In the latter case, bees were trained against their spontaneous preferences (Fig. 5); nevertheless, their performance did not differ from the group rewarded on the higher humidity flowers, which suggests that it is not difficult for bees to learn to favour less-humid flowers if they are more rewarding. Spontaneous preferences of naive bees in favour of humid flowers were not seen in the initial stages of learning experiments (as each group showed similar initial success of 0.5), which suggests that the bees quickly learned floral identity and altered their behaviour.

While there were temperature differences between artificial flower variants, these differences were small (<1°C) and below that required to alone induce these kinds of foraging responses to flowers in bees (Heran, 1952). If bees were responding to a temperature difference alone as opposed to humidity differences, foraging responses comparable to those observed in preference and learning experiments would require greater between-flower temperature differences (4°C or more based upon: Dyer et al., 2006; Harrap et al., 2017; Whitney et al., 2008). Additionally, in the passive artificial flower preference test, we would have expected to find the reverse of the preferences observed (Dyer et al., 2006), as dry passive flowers were slightly warmer (the bees’ temperature preference) than humid variants. Studies of antennal projections in *Drosophila* show that humidity and temperature information might be integrated at the level of the antennal lobe of insects (Frank et al., 2017). We can therefore not fully exclude the possibility that foraging responses are modulated by the small temperature difference. Regardless, this cannot explain the preference we found. Bees showed a preference for elevated floral humidity when presented by both flower types, when humid variants were both very slightly hotter or cooler, suggesting these small differences in temperature did not modify the floral humidity preference response observed here. In learning trials, it is possible this small concurrent temperature difference may slightly enhance pollinator learning (a multimodal interaction) relative to learning if humidity differences presented to bees without these small concurrent temperature differences. However, as learning based on floral temperature differences between flowers requires larger temperature differences between flowers, the learning response here appears to be in response to the humidity differences.

Our results indicate that floral humidity may represent a floral signal or cue that can be used far more widely than previously thought. The hygrosensitive ceolocapitular sensilla of generalist pollinators such as bees are likely to be able to respond to both the amount of humidity produced by the flower itself (Yokohari, 1983; Yokohari et al., 1982) and the rate of change in humidity experienced as the bee approaches or passes the flowers (Tichy and Kallina, 2014, 2010). This ability to distinguish different levels of flower humidity has important consequences, given that natural flowers can differ in the level of floral humidity they produce, with 
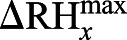
 observed to range between species from 0.05% in *Fuchsia* sp. to 3.71% in *Calystegia sylvatica* (Harrap et al., 2020a). The presence, absence and difference in floral humidity between flowers may function as part of the multisensory displays and transmit valuable information that bumblebees can respond to and learn whilst detecting, choosing or handling flowers. Consequently, traits that influence the floral humidity production may be adaptive to plants (Harrap et al., 2020a). Traits that increase floral humidity levels may increase visitation by naive bumblebees, positively influencing their unlearned preferences and, by creating differences in humidity between flowers, aid the learning and recognition of flowers from those that produce less humidity. Nevertheless, floral traits that suppress floral humidity production may still exist in natural flowers, as these traits can also be adaptive. Although naive bees may be less attracted to flowers with reduced floral humidity, the absence of humidity can be easily learned by pollinators if it represents a predictive cue for floral rewards, as we show here. For example, previous work has shown that *Vinca herbacea* and *Linum grandiflorum* showed no floral humidity or less humidity than extraneous humidity sources (Harrap et al., 2020a). Based on the findings of our learning experiments, we would predict that bumblebees detect such a lack of humidity and would very easily distinguish these flowers from humidity-producing species. Similar adaptations of the floral display that go against naive bee preferences but may enhance floral recognition have been observed previously in non-blue-coloured flowers (Dyer and Chittka, 2004; Gumbert et al., 1999; Lynn et al., 2005) and cold flowers (Whitney et al., 2008). However, plants are likely to be subject to other selective pressures that might have greater influence on the evolution of traits that determine floral humidity production than pollinator responses to floral humidity. For example, as nectar evaporation and transpiration have important contributions to floral humidity production but are also subject to selective pressures associated with limiting water loss (Galen, 1999; Galen et al., 1999; Gallagher and Campbell, 2017; Lambrecht, 2013; Lambrecht and Dawson, 2007) or floral temperature control (Patiño and Grace, 2002; Shrestha et al., 2018), these may have a greater impact on the evolution of a plant species' capacity to produce floral humidity.

Our findings extend the understanding of plant–pollinator interactions but also shed light on a novel function of humidity perception in insects. As an environmental cue for insects (Abou-Shaara et al., 2017; Enjin, 2017), humidity can have important influences on levels of foraging activity (McCall and Primack, 1992; Peat and Goulson, 2005), (micro)habitat selection (particularly when avoiding desiccation) (Enjin, 2017; Knecht et al., 2017; Perttunen and Erkkilä, 1952; Sun et al., 2018), selection of oviposition sites (Okal et al., 2013), locating vertebrate hosts (Chappuis et al., 2013; Olanga et al., 2010; Smart and Brown, 1956), nest maintenance (Human et al., 2006; Nicolson, 2009) and context-dependent flight responses (Wolfin et al., 2018). We show here that this well-developed sensory capacity could also be used to inform foraging and learning of floral displays. Like many insects (Liu et al., 2007), other bees (Fialho et al., 2014) possess hygrosensitive receptors. However, bumblebees in particular have a more widespread distribution of hygrosensitive receptors across their antennae than other bees (Fialho et al., 2014) and this may result in differences in sensitivity that might determine the extent to which different pollinators can make use of floral humidity cues.

It is important to note that, when applying the findings of this study to natural systems, floral humidity cues will be encountered as part of the display of natural flowers. Flowers will usually be embedded in vegetation. The vegetative tissue of plants is also likely to be a source of humidity via transpiration. While floral humidity has been seen to be elevated when measured alongside vegetive tissue within natural systems (Norgate et al., 2010), it is possible that the presence of another proximal humidity source may make floral humidity less distinct, by effectively raising the surrounding ‘background’ humidity. However, many flowers are presented on stalks that might sufficiently spatially separate them from vegetative tissue, and any elevated humidity from it, allowing floral humidity to not compete with vegetative humidity. Also, flowers have additional sources of humidity production, such as liquid nectar (Corbet, 2003; von Arx et al., 2012). Floral tissue is often more permeable than leaves (Buschhaus et al., 2015), allowing greater water loss (Galen et al., 1999; Gallagher and Campbell, 2017; Lambrecht, 2013; Teixido and Valladares, 2014), and the complex three-dimensional structures of flowers, and their greater surface area relative to their headspace, may allow more transpiration to occur within the flower headspace than that of vegetation (Harrap et al., 2020a). Thus, many flowers will remain distinct from the surrounding vegetation in terms of headspace humidity. If vegetation does elevate humidity, regardless of whether individual flowers remain distinct within this, the bee humidity preferences and learning demonstrated in this study may function to guide foraging decisions at the plant or the foraging patch level, as already observed in moths (Wolfin et al., 2018). In this way, humidity responses may allow bees to learn and locate areas of vegetation, which contain plants and flowers for forage within. Groups of humidity-producing flowers occurring together as group displays (be these made up of flowers from different plants or inflorescences of the same plant) may elicit similar patch-level responses.

A related important consideration is that, while we found that bees have the capacity to respond to floral humidity differences comparable to those seen in nature, this does not mean that these are utilised within natural settings. While humidity cues presented to bees in this trial match those produced by flowers (Corbet, 2003; Corbet et al., 1979; Harrap et al., 2020a; von Arx et al., 2012), real flowers present multimodal floral displays that are dominated by visual and olfactory cues (Raguso, 2004; Nordström et al., 2017). The spontaneous floral humidity preferences of naive bumblebees were subtle (Fig. 5), despite the differences in humidity between the artificial flowers being comparable to the larger differences observed between natural flowers (Harrap et al., 2020a). In the presence of these more salient modalities to which bees have stronger preferences and are perhaps more likely to attend, such as colour and scent, responses to humidity cues may be superseded. However, experimental studies are increasingly showing that pollinators, particularly bees, are able to utilise ‘additional’ floral cues presented as part of multimodal displays (Leonard et al., 2011a, 2012; Leonard and Masek, 2014). As we have demonstrated here, bees have the capacity to respond to floral humidity, and it is therefore possible that humidity may be responded to in kind as part of a flower's multimodal display.

Within multimodal displays, different cues may have different roles (Leonard et al., 2012). Because floral humidity has strong links to nectar evaporation, floral humidity has been proposed as an ‘honest signal’ (von Arx, 2013; von Arx et al., 2012). In *O. caespitosa* flowers, removal or blocking of floral nectar reduced the intensity of floral humidity. Honest signals correspond with the reward state of flowers, indicating temporary rewardlessness to pollinators (e.g. as a result of a recent visitation by a pollinator), allowing pollinators to avoid wasteful visits to unrewarding flowers, and increasing pollinator efficiency and preference to honest signallers (Knauer and Schiestl, 2015; von Arx, 2013). The spontaneous and learned responses to floral humidity demonstrated here may allow bumblebees to adjust visitation to favour rewarding flowers. However, it is uncertain whether floral humidity intensity directly indicates flower reward state, and therefore functions honestly, in all species that produce it.

Presentation of ‘additional’ cues such as floral electrostatics (Clarke et al., 2013) and temperature (Harrap et al., 2020b), alongside visual and scent cues as part of multimodal displays, has been demonstrated to enhance pollinator responses relative to responses to individual modalities, as has the presentation of visual cues alongside scent (Kaczorowski et al., 2012; Katzenberger et al., 2013; Kunze and Gumbert, 2001). In particular, when flowers present multiple modalities, bees can learn and distinguish flowers to a greater degree of accuracy and more quickly (Clarke et al., 2013; Katzenberger et al., 2013; Kulahci et al., 2008), even when these additional cues do not differ with that initial signal (Kunze and Gumbert, 2001; Leonard et al., 2011b). This may be due to additional cues allowing displays to be more distinct, or having additional functions contextualising other signals or changing perceptual salience when presented together. In this same manner, the presence of floral humidity cues that pollinators can detect might enhance pollinator preferences and learning of multimodal floral displays (irrespective whether floral humidity directly corresponds with reward state, functioning as an ‘honest cue’). The presence of neurological or perceptual links between modalities appears important in determining how bees respond to certain multimodal combinations of cues (Harrap et al., 2019, 2020b; Lawson et al., 2017b, 2018). Furthermore, flower processes involved in temperature regulation such as transpiration (Patiño and Grace, 2002; Shrestha et al., 2018) are also involved in humidity generation (Corbet et al., 1979; Harrap et al., 2020a; von Arx et al., 2012), and so pollinators may frequently encounter humidity and temperature cues together. Now that the capacity to respond to biologically relevant levels of floral humidity has been demonstrated here in a widespread pollinator species, we suggest that further investigation should be conducted into how floral humidity is used as part of a multimodal display, which should help to provide a more holistic understanding of plant–pollinator interactions in nature and the influence of floral humidity within these.

## Supplementary Material

Supplementary information
