# Supplementary material for: Bumblebees can detect floral humidity
Source: J Exp Biol. 2021 Jun 24;224(12):jeb240861. doi: 10.1242/jeb.240861 (PMC8246344; doi:10.1242/jeb.240861)
Supplement: Supplementary information [file jexbio-224-240861-s1.pdf]

Table S1. The best fitting models, X_t^{max} and ΔRH_x^{max} values for both variants of both types of artificial flowers. Subscript letters following best fitting models indicate shape of that model: L, linear models; and Q, quadratic models (note quadratic models were not fitted to the z axis). Subscript values next to X_t^{max} values indicate replicate effects: the number itself referring to the replicate transect at which ΔRH_x^{max} was found; no subscript values indicates replicate transects have no effect on humidity. For further details on models, see Harrap et al. (2020) and the code attached to the datafiles of this publication (Harrap et al., 2021). For AIC tables pertaining to humidity structure model selection, see supplementary materials and methods 1.

Artificial Flower Type	Flower variant	Best fitting model for the *x* axis	Best fitting model for the *z* axis	X_t^{max}	ΔRH_x^{max}
Active	Humid	$m3_Q$	$z1_L$	2.19	3.08
	Dry	$m10_Q$	$z4_L$	-0.12_1	0.92
Passive	Humid	$m9_Q$	$z3_L$	0_1	3.49
	Dry	$m10_Q$	$z4_L$	-0.61_1	2.13

Table S2. The parameter values of the best fitting models of both *x* and *z* axis models from our analysis of humidity structure of each variant of each artificial flower type. Parameters are identified by the R model fixed effect labels as used in the code attached to the datafiles of this publication (Harrap et al., 2021), column 'R', and the parameter names given for the equivalent parameters in Harrap et al. (2020), column ID, to facilitate comparison. For further detail on parameters of models and parameter function consult Harrap et al. (2020, 2021). All values are given in scientific format (g Ex = $g \cdot 10^x$).

Flower type		Active flowers		Passive flowers	
Flower Variant		Humid	Dry	Humid	Dry
ID	*R*				
I_x	(intercept)	3.07 E+00	9.25 E-01	3.49 E+00	2.13 E+00
A_x	*xoffset*	1.48 E-02	-2.96 E-04		-3.50 E-03
B_x	*x2*	-3.38 E-03	-1.26 E-03	-4.05 E-03	-2.85 E-03
r_{2x}	*rep1*		-8.52 E-01	-1.01 E-01	-4.89 E-01
r_{3x}	*rep2*		-8.52 E-01	-6.91 E-01	-9.35 E-01
r_{4x}	*rep3*		-9.34 E-01	-9.11 E-01	-1.28 E+00
g_{2x}	*xoffset:rep1*		-2.64 E-03		-2.24 E-03
g_{3x}	*xoffset:rep2*		-1.98 E-03		-2.26 E-03
g_{4x}	*xoffset:rep3*		-2.23 E-03		-1.40 E-03
c_{2x}	*x2:rep1*		1.06 E-03	3.17 E-04	7.83 E-04
c_{3x}	*x2:rep2*		1.18 E-03	1.16 E-03	1.60 E-03
c_{4x}	*x2:rep3*		1.20 E-03	1.51 E-03	1.92 E-03
I_z	(intercept)	2.01 E+00	4.04 E-01	1.27 E+00	9.39 E-01
B_z	*lnzoffset*	-5.50 E-01	-1.12 E-01	-3.86 E-01	-3.16 E-01
r_{2z}	*rep1*		-5.87 E-01	1.17 E-01	-2.32 E-01
r_{3z}	*rep2*		-5.89 E-01	1.93 E-01	-3.68 E-01
r_{4z}	*rep3*		-5.96 E-01	2.17 E-01	-7.36 E-01
c_{2z}	*lnzoffset:rep1*		1.76 E-01		1.09 E-01
c_{3z}	*lnzoffset:rep2*		2.01 E-01		1.97 E-01
c_{4z}	*lnzoffset:rep3*		1.71 E-01		2.87 E-01

Journal of Experimental Biology • Supplementary information

Table S3. AIC tables and sampling dates of artificial flower floral humidity analyses

For each individual artificial flower of each variant the date and time at which the first x axis transect replicate began is given (YYYY-MM-DD-hh-mm-ss). In each AIC table, each species having one for x and z axis models, AIC and degrees of freedom ‘df’ are given: see (Harrap et al., 2020) for description of the different models. Difference in ΔAIC, here calculated as AIC of model with the lowest AIC minus that of the current model, is also provided. Within each AIC table, shaded and in bold are the best fitting models as per the guidelines given in (Richards, 2008).

Active Humid

X axis model	df	AIC	ΔAIC
**m3**	**5**	**1114.88**	**0.00**
m7	8	1119.73	-4.85
m10	14	1127.27	-12.39
m2	4	1151.32	-36.44
m6	7	1156.27	-41.39
m9	10	1158.71	-43.82
m1	4	1473.78	-358.90
m5	7	1479.30	-364.42
m8	10	1485.08	-370.20
m0	3	1488.09	-373.21
m4	6	1493.63	-378.75

Z axis model	df	AIC	ΔAIC
**z1**	**4**	**86.67**	**0.00**
z3	7	92.09	-5.42
z4	10	95.24	-8.57
z0	3	233.29	-146.62
z2	6	239.03	-152.36

Sampling dates	
	2017-11-16-11-08-52
	2017-11-16-12-26-15
	2017-11-16-13-43-38
	2017-11-16-15-01-05
	2018-02-01-11-54-27
	2018-02-01-10-37-04
	2018-02-01-13-11-52
	2018-02-01-14-29-17

Active Dry

X axis model	df	AIC	ΔAIC
**m10**	**14**	**-29.10**	**0.00**
m9	10	-22.24	-6.85
m7	8	126.84	-155.94
m6	7	132.75	-161.84
m5	7	205.87	-234.96
m8	10	210.19	-239.28
m4	6	210.35	-239.45
m3	5	314.15	-343.25
m2	4	317.09	-346.18
m1	4	364.45	-393.55
m0	3	366.80	-395.90

Z axis model	df	AIC	ΔAIC
**z4**	**10**	**-347.76**	**0.00**
z3	7	-312.58	-35.18
z2	6	-310.97	-36.78
z1	4	-279.22	-68.53
z0	3	-278.31	-69.44

Sampling dates	
	2017-11-20-15-10-07
	2017-11-20-16-27-30
	2017-11-20-17-44-55
	2017-11-20-19-02-19
	2018-01-30-10-35-56
	2018-01-30-11-53-23
	2018-01-30-13-10-46
	2018-01-30-14-28-06

Passive Humid

X axis model	df	AIC	ΔAIC
**m9**	**10**	**1208.27**	**0.00**
m10	14	1214.90	-6.62
m6	7	1260.35	-52.08
m7	8	1261.82	-53.54
m2	4	1298.20	-89.93
m3	5	1299.70	-91.43
m4	6	2047.90	-839.63
m5	7	2049.75	-841.48
m0	3	2054.29	-846.01
m8	10	2055.56	-847.28
m1	4	2056.14	-847.87

Z axis model	df	AIC	ΔAIC
z4	10	-125.11	0.00
**z3**	**7**	**-122.95**	**-2.15**
z1	4	-76.13	-48.98
z2	6	111.63	-236.74
z0	3	129.54	-254.64

Sampling dates	
	2017-10-03-10-34-17
	2017-10-03-13-09-02
	2017-10-04-10-58-13
	2017-10-04-12-15-36
	2017-10-05-11-36-06
	2017-10-05-14-10-52
	2017-10-10-13-23-13
	2017-10-10-14-40-36
	2017-10-23-12-10-47
	2017-10-23-13-28-10
	2017-10-24-10-17-19
	2017-10-24-12-52-05

Passive Dry

X axis model	df	AIC	ΔAIC
**m10**	**14**	**872.95**	**0.00**
m9	10	890.60	-17.65
m7	8	1001.48	-128.54
m6	7	1019.29	-146.34
m3	5	1102.00	-229.05
m2	4	1116.68	-243.74
m5	7	1468.42	-595.48
m8	10	1474.10	-601.16
m4	6	1475.71	-602.76
m1	4	1514.24	-641.29
m0	3	1520.78	-647.83

Z axis model	df	AIC	ΔAIC
**z4**	**10**	**-156.34**	**0.00**
z3	7	-131.26	-25.08
z1	4	-103.48	-52.86
z2	6	-67.79	-88.56
z0	3	-46.80	-109.54

Sampling dates	
	2017-10-03-11-51-40
	2017-10-03-14-26-27
	2017-10-04-13-32-59
	2017-10-04-14-50-22
	2017-10-05-10-18-43
	2017-10-05-12-53-29
	2017-10-10-10-48-25
	2017-10-10-12-05-48
	2017-10-23-10-53-22
	2017-10-23-14-45-33
	2017-10-24-11-34-42
	2017-10-24-14-09-28

Journal of Experimental Biology • Supplementary information

References in Supplementary Information

Harrap, M.J.M., Hempel de Ibarra, N., Whitney, H.M., Rands, S.A., 2020. Floral humidity in flowering plants: a preliminary survey. Front Plant Sci 11, 249. https://doi.org/10.3389/fpls.2020.00249

Harrap, M.J.M., Knowles, H.D., Hempel de Ibarra, N., Whitney, H.M., Rands, S.A., 2021. Data from "Bumblebees can detect floral humidity." Figshare Database. https://doi.org/10.6084/m9.figshare.14292320

Richards, S.A., 2008. Dealing with overdispersed count data in applied ecology. J Appl Ecol 45, 218–227. https://doi.org/10.1111/j.1365-2664.2007.01377.x

Journal of Experimental Biology • Supplementary information
